# Right heart thrombus in transit and peripherally inserted central catheters

**DOI:** 10.1186/s12959-023-00513-3

**Published:** 2023-06-22

**Authors:** Rezwan N Hussain, Amit K J Mandal, Nick Li, Jihène El Kafsi, Anastasis Sioftanos, Constantinos G Missouris

**Affiliations:** 1 Wexham Park Hospital, Frimley Health NHS Foundation Trust, Slough, UK; 2 The Queen’s College, University of Oxford, Oxford, UK; 3 Ashford and St Peter’s NHS Foundation Trust, Chertsey, UK; 4 University of Nicosia Medical School, Nicosia, Cyprus

**Keywords:** Right heart thrombus, Peripherally inserted central catheters, PICC, Thrombosis, Risks, Imaging, Anticoagulation

## Abstract

Right heart thrombus in transit or “free-floating right heart thrombus” is defined as thrombus(i) arising from the deep veins that embolises to the right atrium or right ventricle before reaching the pulmonary vasculature. It is almost always associated with pulmonary thromboembolism and is a medical emergency with reported mortality rates of over 40%. We present two cases of right heart thrombus in transit with pulmonary thromboembolism resulting from venous thrombosis associated with peripherally inserted central catheters that were managed with different approaches. The cases highlight that clinicians should have a low threshold to utilise imaging modalities such as computerised tomography and transthoracic echocardiography when there is an untoward change in physiological parameters among patients with peripherally inserted central catheters, particularly those with risk factors for peripherally inserted central catheter associated venous thrombosis. Furthermore, procedural optimisation surrounding peripherally inserted central catheters, such as insertion technique and choice of lumen size, is underscored.

**Supplementary Information:**

The online version contains supplementary material available at 10.1186/s12959-023-00513-3.

## Case 1

**Fig. 1:**
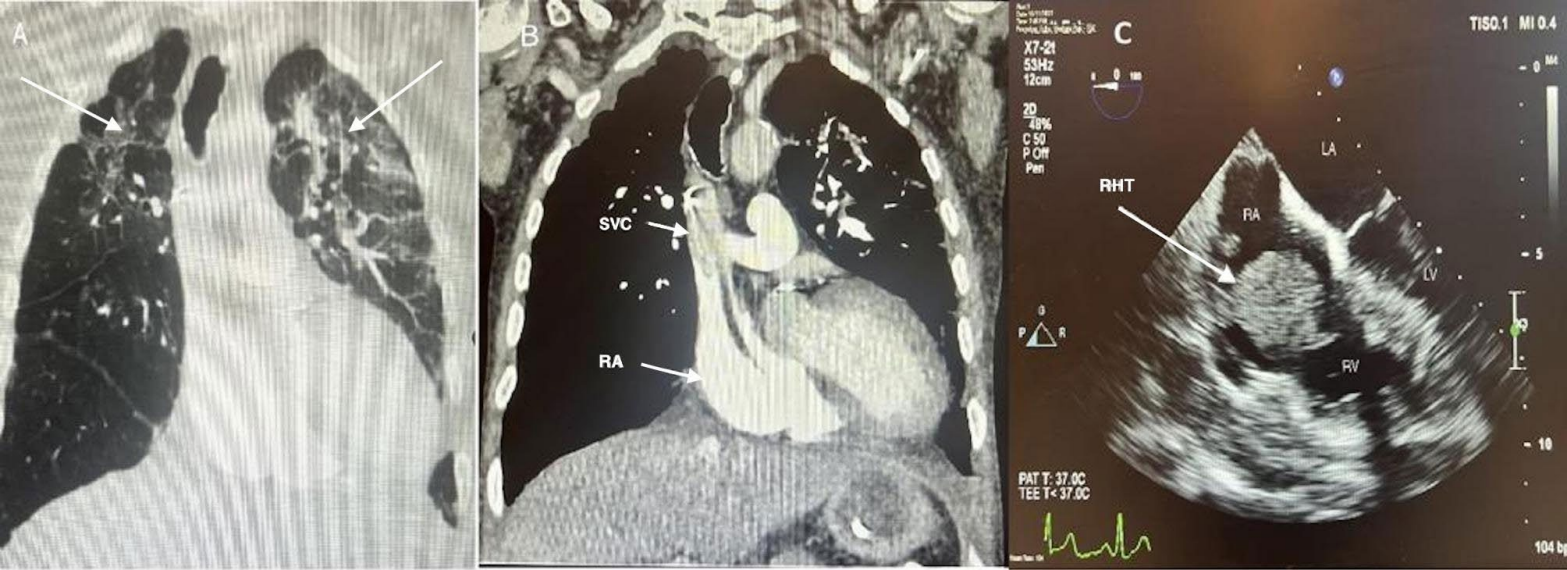
**a**: CT chest (coronal section) demonstrating bilateral upper and middle zone parenchymal changes; **b**: CTPA (coronal section) demonstrating thrombus in transit extending into the right atrium (arrows); **c**: TTE 4 chamber view demonstrating mobile thrombus in the RA prolapsing through the TV

A 77-year-old Caucasian male presented with a 6-month history of cough and unintentional weight loss. Clinical examination revealed emaciation. Computerised tomography (CT) chest demonstrated bilateral upper and middle zone parenchymal changes (Fig. 1a). Laboratory investigations were notable for C-reactive protein (CRP) of 100 mg/L and iron deficient anaemia (haemoglobin (Hb) of 83 g/L and transferrin saturation of 6%). Sputum culture tested positive for acid-fast bacilli (AFB) and the patient was initiated on anti-tuberculous treatment. Over the next week the patient developed an acute abdomen and CT demonstrated pneumoperitoneum. At laparotomy a large pelvic abscess with ileal perforation was evident and a double barrel ileostomy was formed. Purulent material was positive for AFB.

Standard quintuple anti-tuberculous regimen and parenteral nutrition were administered via a triple lumen 5 French (Fr) peripherally inserted central catheter (PICC) inserted into the right cephalic vein which had been sited using ultrasound guided micropuncture technique. Placement of the catheter tip in the lower third of the superior vena cava (SVC) was confirmed using plain chest radiography. There was no previous vascular access history. 5 days later the patient suddenly became profoundly hypoxic and CT pulmonary angiography (CTPA) demonstrated the catheter tip in the SVC with thrombus in the right brachiocephalic vein extending into the right atrium (RA) (Fig. 1b). The patient was prescribed unfractionated heparin (5,000 units given as an intravenous bolus then infusion of 30,000 units over 24 h with the rate adjusted according to activated partial thromboplastin time (APTT) ratio [range 1.5 to 2.5]). Transthoracic echocardiography (TTE) demonstrated large, mobile thrombus in the RA prolapsing through the tricuspid valve (TV) (Fig. 1c and video 1). On repeat TTE 2 days later, the absence of thrombus in the right heart suggested the dispersal to the pulmonary circuit. The patient remained physiologically stable and oxygen was weaned after 3 days.

Due to large fluctuations in APTT ratio, unfractionated heparin was switched to low molecular weight heparin (dalteparin with weight adjusted dose of 7,500 units admisnistered subcutaneously once daily) after 1 week and warfarin was initiated after 2 weeks with a target international normalised ratio (INR) of 2–3.

Over the subsequent 3 weeks, with a therapeutic INR, the patient developed epistaxis and haematuria with clot retention. Warfarin was therefore substituted for dalteparin indefinitely. The patient was discharged 6 months after admission in a good condition having completed anti-tuberculous treatment. Repeat TTE demonstrated normal cardiac structure and function without intracardiac thrombus.

## Case 2

A 58-year-old woman presented with rigors and tenderness at the insertion site of a PICC in the right arm. A single lumen 4 Fr PICC had been sited in the basilic vein using ultrasound guided micro-puncture technique 13 weeks previously. Chest radiogaraph at the time confirmed correct placement of the catheter tip in the lower third of the SVC. Clinical examination revealed tachycardia, tachypnoea, hypoxia and an erythematous, swollen right upper limb. She had just completed adjunct chemotherapy (4 cycles of carboplatin and paclitaxel) after total abdominal hysterectomy and bilateral salpingo-oophorectomy for stage 1 A differentiated carcinoma of the uterus. Medical history was also significant quiescent Beçhet’s disease, for which she was prescribed long term treatment with hydroxychloroquine, and treatment for deep vein thrombosis (DVT) 20 years ago. Vascular access history was otherwise unremarkable. Laboratory investigations were notable for CRP of 188 mg/L and Hb of 64 g/L with associated iron deficiency. Venous doppler ultrasound revealed acute thrombus in the basilic vein extending to the axillary and subclavian veins. The PICC was removed and dalteparin was administered. Blood cultures yielded Staphylococcus aureus and the patient was prescribed intravenous flucloxacillin. The patient also received transfusions of packed red blood cells.

**Fig. 2:**
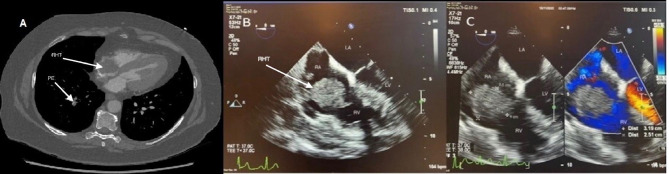
**a**: CTPA (sagittal section) demonstrating RHT and segmental PE (arrows); **b, c**: TOE (4 chamber view) showing giant RHT prolapsing through the TV in diastole

CTPA demonstrated a thrombosed axillary vein and thrombus in transit within the RA and bilateral pulmonary thrombi (Fig. 2a). The catheter tip was encased in clot, and located in the SVC. TTE and transoesophageal echocardiography (TOE) revealed multiple masses in the RA, with the largest clot measuring 4 × 3.5 cm (Fig. 2b and c, Video 2).

The patient was anticoagulated with dalteparin 18,000 units subcutaneously once daily and remained haemodynamically stable maintaining oxygen saturations at 96% on room air. Due to the large size of the thrombus, mechanical thrombectomy was impossible and the patient was transferred to a tertiary cardiac centre and underwent uncomplicated surgical thrombectomy 22 days after presentation. During the post-operative period she was supratherapeutically anticoagulated with dalteparin 10,000 units twice daily on haematology advice, and after discharge established on life-long therapy with rivaroxaban 20 mg daily.

## Discussion

PICCs are widely inserted in clinical practice for the administration of chemotherapy, antibiotics, parenteral nutrition, haematopoietic stem cell transplantation and blood transfusion [1, 2]. Most common complications associated with PICCs are venous thromboembolism (VTE), infection, accidental removal, line fracture and embolisation [1]. The risk of PICC associated DVT (PICC-DVT) is heightened with high body mass index, thrombocytosis, cancer and chemotherapy, inflammatory conditions, infections, recent surgery, smoking, characteristics of the catheter such as size, type, tip location, insertion site, and retention time. PICC-DVT increases mortality and morbidity in cancer patients [3].

One mechanism in which PICCs may induce thrombosis is through reducing blood flow in the indwelling vein. Ex-vivo models have shown that PICCs may cause a reduction in flow by as much as 93%, and indeed, the catheter-to-vein size ratio is a significant risk factor for PICC-DVT [4–6]. Furthermore, the thrombogenic nature of the catheter itself, the endothelial disruption caused by insertion and continued agitation whilst in situ have been postulated as possible causative explanations for PICC-DVT [7]. As such, PICC-DVT may be considered a direct consequence of reduced blood flow, PICC insertion and intimal injury, the risk being increased by other systemic factors.

Once PICC-DVT is established, development into RHT or PTE is rare. However, the presence of RHT complicating PTE carries with it an increased mortality rate compared to PTE alone. The incidence of RHT associated with central venous catheters is reported at 29% in post-mortem studies [8] and is associated with a mortality of over 40% [9]. Individual case reports have implicated 2 distinct pathophysiological explanations for the formation of catheter related RHT: extension of in-situ thrombosis [10]; or embolization from PICC-DVT related to the site of insertion [11]. In our case series, CT angiography clearly demonstrated direct extension of in-situ thrombosis.

In a study of ambulating cancer outpatients, thromboprophylaxis with direct oral anticoagulants did not decrease the incidence of PICC-DVT [7]. There is a need for further research to evaluate the optimum VTE chemoprophylaxis within the context of PICC insertion, particularly for patients at augmented risk. At the time of writing, anticoagulation with low molecular weight heparin or vitamin K agonists for a minimum of 3 months is accepted treatment for PICC-DVT, despite any specific randomised controlled trials. In addition, it is unclear whether PICCs may remain in situ when functional and clinically necessary in the presence of PICC-DVT [12–14]. Despite new evidence surrounding standard of care for patients with VTE, there remains substantial uncertainty [15, 16].

Morphologically, RHT has been classified into types: A and B. Type A, or ‘thrombus(i) in transit’ (as in our 2 cases) originate in the peripheral veins and extend or embolise to the right heart chambers, particularly the RA. These thrombi are mobile and can prolapse through the TV. The association of free-floating RHT and massive PTE has an incidence of 4-18% [17]. Type B thrombi are attached to the RA or right ventricle (RV) wall and may develop in conjunction with intracardiac foreign objects (such as pacing wires or intravascular catheters) or in chambers with structural abnormalities [18]. Type B thrombi are less mobile, often polypoid with a broader base, less likely to disperse into the pulmonary circuit and, therefore, carry a better prognosis [19].

Management techniques for hemodynamically significant PTE have been well documented, but the optimal therapeutic approach for RHT with and without PTE is unclear. However, timely catheter removal and expeditious systemic anticoagulation seems logical [16, 20].

Kajamohideen et al. analysed 177 cases of RHT. Treatments administered were none (9%), anticoagulation therapy (35.0%), surgical procedure (35.6%), or thrombolytic therapy (19.8%). The overall mortality rate was 27.1%. The mortality rate associated with no therapy, anticoagulation therapy, surgical embolectomy, and thrombolysis was 100.0%, 28.6%, 23.8%, and 11.3%, respectively [21]. Favourable outcomes with systemic thrombolysis have been reported in a small series of type A RHT but debate has arisen given the inherent risk of fragmentation and cardiogenic shock in patients with larger clots [19, 22]. Thrombolysis also carries the risk of catastrophic bleeding. Mechanical (percutaneous) or surgical thrombectomy is pursued when medical therapy fails or if there is a contraindication to thrombolysis in the context of dynamic instability. Surgical thrombectomy is indicated for larger volume thrombus as seen in our second patient. However, this requires full cardiopulmonary bypass and is sometimes associated with an extremely high mortality rate [23]. Furthermore, it is not readily available in all centres.

TTE should be performed in hemodynamically unstable patients with central catheters to assess for RV enlargement and dysfunction as well as RHT. RHT can cause RV failure particularly when there is TV or pulmonary valve obstruction. TOE may be preferred over TTE in the evaluation of RHT due to its improved ability to identify and describe the morphology and size of clots and their suitability for mechanical or surgical thrombectomy.

Patient related risk factors for PICC-DVT have been well described among cancer patients [24, 25]. Both our patients had several risk factors for the development of PICC-DVT and RHT. The first patient had a diagnosis of active pulmonary/extra-pulmonary tuberculosis and had undergone recent major surgery. For this patient, the only safe management was systemic anticoagulation. Thrombolysis was contraindicated after recent surgery. Thankfully, the patient remained haemodynamically stable but percutaneous thrombectomy would have been considered. The patient’s frailty and cachexia would have put him at high risk for complications from cardiopulmonary bypass and an open procedure. The case also highlights the complications of systemic anticoagulation in a polymorbid, sarcopaenic and frail patient, and that anticoagulation regimens need to be tailored on an individual basis. Our second patient had multiple risk factors for PICC-DVT: recent chemotherapy for uterine cancer, Beçhet’s disease, a protracted PICC retention period and a past medical history of DVT. Thrombolysis would have been considered if the patient had become haemodynamically compromised, albeit with an inherent risk of clot fragmentation. A percutaneous approach was discussed, but due to the giant size of the thrombus and generally good and stable condition of the patient, we decided to proceed with surgical management, which was successful and uncomplicated.

Whilst patient related risk factors are usually non-modifiable, technical factors such as insertion technique and choice of lumen size are very important and should be optimised to reduce the risk of PICC-DVT. A catheter to vessel ratio is recommended to assist clinicians in selecting the most appropriate sized device for the vessel, and PICCs should not exceed 45% of the cross-sectional area of the cannulated vein [26, 27]. Despite the association of RHT and PTE extending from PICC-DVT described in these two cases, PICCs remain largely safe to use and such cases are rare [28].

## Conclusion

Technical factors surrounding the use and insertion of PICCs should be optimised based on individual clinical considerations to minimise the risk of PICC-DVT. The development of RHT in transit is rare phenomenon with potentially fatal pulmonary migration. A high index of suspicion for PICC-DVT and clot propagation should be exercised in patients with PICCs and thrombogenic states, such as underlying malignancy, chronic infective and inflammatory syndromes. Clinicians should have a low threshold to utilise imaging modalities such as CT and TTE when there is an untoward change in physiological parameters.

## Electronic supplementary material

Below is the link to the electronic supplementary material.

Supplementary Material 1: Video 1 TTE (4 chamber view) demonstrating mobile RHT in the RA prolapsing through the TV.

Supplementary Material 2: Video 2 TOE (4 chamber view) demonstrating giant RHT prolapsing through the TV in diastole.

## Data Availability

All data and materials are available from the corresponding author upon reasonable request.
